# The Pattern of Alcohol Consumption and the Severity of Alcohol-related Liver Disease in Patients Visiting the Liver Clinic

**DOI:** 10.7759/cureus.7251

**Published:** 2020-03-12

**Authors:** Shoukat Ali Samjo, Zaigham Abbas, Muhammad Asim, Kanwal Tahir

**Affiliations:** 1 Gastroenterology and Hepatology, Ziauddin University Hospital, Karachi, PAK; 2 Gastroenterology, Ziauddin University Hospital, Karachi, PAK

**Keywords:** alcohol-related liver disease, alcohol misuse, alcohol-related cirrhosis

## Abstract

Introduction

The purpose of this study was to discern the pattern of alcohol consumption and the severity of alcohol-related liver disease (ARLD) in patients visiting the tertiary care hospital.

Methods

A cross-sectional study was conducted at Dr. Ziauddin Hospital Clifton campus, Karachi. Patients visiting the liver clinic with disturbed liver enzymes and a history of alcohol intake after excluding other causes were included. A detailed history, routine investigations, insulin level, abdominal ultrasound, and transient elastography were performed.

Results

A total of 155 patients were included in the study, 98% of whom were men. The median age was 45.93 years (range: 18-78 years). Just over three-fourths of the visiting patients were Muslim (n=119; 76.8%). The median duration of alcohol intake was 5.7 years. All patients admitted to consuming alcohol on an empty stomach before dinner. The most common associated addiction was smoking (n=95; 61.2%). Around two-thirds of patients confessed to binge drinking (n=66; 42.9%). According to the Diagnostic and Statistical Manual of Mental Disorders criteria, 92 patients (59.35%) were alcohol dependent. Hepatic steatosis was positively correlated with the weight of patients (p=0.035). Other factors positively correlated with hepatic steatosis included insulin resistance (p=0.031), elevated uric acid levels (p=0.003), and units of alcohol intake (p=0.054). Significant fibrosis (F3-F4) was present in 73 (47.09%) patients. It was correlated with low platelet count, total bilirubin, aspartate aminotransferase, gamma-glutamyl transpeptidase, alkaline phosphatase, international normalized ratio, albumin, uric acid, controlled attenuation parameter, and units of alcohol intake with significant p-values. Further multivariant analysis showed liver fibrosis was correlated with cholesterol level with a significant p-value (p=0.045).

Conclusion

ARLD is mainly a male-dominant disease in our population. Most patients consumed a large volume of highly concentrated alcohol and were alcohol dependent. Insulin resistance was observed in a significant number of patients.

## Introduction

Excessive alcohol consumption is a major healthcare concern around the world. A high level of alcohol intake over decades affects nearly every organ in the body. However, as the liver is the primary site of ethanol metabolism, it suffers the greatest amount of tissue injury [1]. The severity and prognosis of alcohol-related liver disease (ARLD) depend on the amount, pattern, and duration of alcohol consumption. Additional contributing factors include the presence of liver inflammation, diet, genetic predisposition, and the nutritional status of an individual. The amount of alcohol consumed is directly associated with liver disease and associated mortality. The National Institute on Alcohol Abuse and Alcoholism defines a standard drink as 11-14 g of alcohol, one glass of wine, or one 0.33-l (12-ounce) beer [2]. Chronic alcohol consumption leads to a varied spectrum of hepatic lesions, including steatosis, hepatitis, and fibrosis/cirrhosis. Although steatosis is an almost completely benign disease, liver cirrhosis is associated with high rates of morbidity, mortality, and shortening of life expectancy [3]. An association between heavy alcohol consumption and liver disease was recognized more than 200 years ago [4]. Long-term heavy alcohol use is a very common cause of illness and death from liver disease in several countries. Globally, mortality due to liver cirrhosis is highest in France and Spain (over 30 deaths per a population of 100,000 per year) and the lowest in northern European countries (up to five deaths per 100,000 inhabitants per year). Mortality secondary to liver cirrhosis in these countries is in direct proportion to absolute alcohol consumption per capita [5]. Steatosis is the earliest and most common response that develops in more than 90% of problem drinkers who consume four to five standard drinks per day over decades [6,7]. However, steatosis also develops after binge drinking, defined as the consumption of four to five drinks in two hours or less. If the affected individual ceases drinking, steatosis is a reversible condition with a good prognosis. Cirrhosis, on the other hand, is a permanent condition and can lead to grave consequences.

The aim of the study was to discern the pattern of alcohol consumption in patients with ARLD in the local population.

## Materials and methods

A cross-sectional study was conducted at Dr. Ziauddin University Hospital in Clifton, Karachi. Patients visiting the liver clinic were recruited via a non-probability consecutive sampling technique. Patients aged 18 years or older, of either gender, who were found to have a history of alcohol intake with clinical, biochemical, and ultrasound evidence of liver disease were included in the study. Exclusion criteria included pregnant women and patients with non-alcoholic fatty liver disease and secondary causes of hepatic steatosis or fibrosis, such as Wilson’s disease, autoimmune liver disease, gastric bypass surgery, positive serology for hepatitis B or C virus, or drug-induced hepatic steatosis, including methotrexate, tamoxifen, amiodarone, and nucleoside analogs. Ethical approval was received from the hospital’s Ethical Review Committee prior to the study, and written consent was obtained from each participant. The sample size calculation was based on a previous study, which noted alcohol-related liver fibrosis in 24% of the population [8].

Details were recorded related to the daily amount of alcohol ingested by the participants, the type of alcoholic beverage consumed, drinking patterns, and the associated severity of the liver disease. Patients who had fatty liver were further evaluated by anthropometric measurements, metabolic profile, insulin resistance, and transient elastography to determine the degree of fibrosis and steatosis in relation to alcohol intake.

Data were analyzed by IBM SPSS Statistics for Windows, Version 21.0 (IBM Corp., Armonk, NY). Mean and standard deviation were calculated for all continuous variables. Frequency and percentage were calculated for qualitative observations, and chi-square tests and Fisher exact tests were applied where relevant. Correlations were performed by the Pearson correlation test. Regression analysis was conducted to determine independent variables. A p-value ≤0.05 was considered significant.

## Results

A total of 155 patients with ARLD visited the liver clinic, 98% of whom were male. The median age was 45.93 years (range: 18-78 years). Just over three-fourths of the visiting patients were Muslim (n=119; 76.8%), followed by Hindu (n=32; 20.7%) and Christian (n=4; 2.6%). The median duration of alcohol intake was 5.7 years. All patients except two were consuming a 40% to 44% concentration of alcohol. All patients admitted to consuming alcohol on an empty stomach before dinner. Other baseline characteristics of the study population are detailed in Table 1.

**Table 1:** Baseline characteristics of study subjects (N=155) Data are shown as numbers (percentage) or mean±standard error of mean. BMI, body mass index; HB, hemoglobin; TLC, total lymphocyte count; ALT, alanine aminotransferase; AST, aspartate aminotransferase; GGT, gamma-glutamyl transpeptidase; ALP, alkaline phosphatase; PT, prothrombin time; INR, international normalized ratio; CR, creatinine; HDL, high-density lipoprotein; LDL, low-density lipoprotein; VLDL, very low density lipoprotein; HOMA-IR, homeostasis model assessment of insulin resistance.

Variable	Mean±SD	Range
Age	45.93±12.052	18-78
Height (cm)	172.5±13.2	64-198
Weight (kg)	84.4±14.2	55-170
BMI (kg/m^2^)	27.5±3.5	17.7-45.1
Waist (cm)	90.5±8.3	76-138
HB (g/dL)	12.2±1.86	7.9-17.0
TLC (x10^9^/L)	6.2±2.5	0.9-14.0
Platelets (x10^9^/L)	198.7±84.18	20-458
Total bilirubin (mg/dL)	1.87±3.01	0.1-23
ALT (IU/L)	61.3±64.5	19-501
AST (IU/L)	57.9±59.8	10.3-425.0
GGT (IU/L)	189.6±326.2	19.0-2500
ALP (IU/L)	130.4±132.5	45.0-989
PT	16.3±4.1	10.0-37.0
INR	1.3±0.4	0.8-3.5
CR (mg/dL)	0.96±0.21	0.20-2.6
Cholesterol (mg/dL)	190.5±31.0	107.0-286.0
Triglyceride (mg/dL)	163.9±61.3	92-522
HDL (mg/dL)	30.4±11.4	20-59
LDL (mg/dL)	96.7±19.9	33-167
VLDL (mg/dL)	77.09±66.06	7.0-85.8
Uric acid (mg/dL)	5.3±2.5	3.5-9.8
Albumin (g/L)	3.5±0.6	1.0-4.8
Insulin (mIU/L)	10.7±3.0	4.0-19.0
HOMA-IR	2.7±1.4	1.0-14.1

The most common associated addiction was smoking (n=95; 61.2%), followed by hashish (n=12; 7.7%). An extramarital sexual affair was admitted by 25 patients (16.5%). Approximately two-thirds of patients reported binge drinking (n=66; 42.09%). According to the Diagnostic and Statistical Manual of Mental Disorders criteria, 92 patients (59.35%) were alcohol dependent. Associated comorbid diseases included diabetes (18; 11.6%), hypertension (n=17; 10.9%), and ischemic heart disease (n=5; 3.2%). Hypercholesterolemia was present in 101 patients (65.2%) and hypertriglyceridemia was present in 98 patients (63.2%). Among 146 patients with fatty liver, 141 patients had elevated alanine aminotransferase levels (steatohepatitis). Hepatic steatosis was positively correlated with the weight of patients (p=0.035). Other factors positively correlated with hepatic steatosis included insulin resistance (p=0.031), elevated uric acid levels (p=0.003), and units of alcohol intake (p=0.054). Insulin resistance was calculated by the homeostatic model assessment of insulin resistance (HOMA-IR) score. Significant fibrosis (F3-F4) was present in 73 patients (47.9%). It was correlated with low platelet count, total bilirubin, aspartate aminotransferase, gamma-glutamyl transpeptidase, alkaline phosphatase, international normalized ratio, albumin, uric acid, controlled attenuation parameter, and units of alcohol intake with a significant p-value, as mentioned in Table 2.

**Table 2:** Factors associated with increased elasticity Data are shown as numbers (percentage) or mean±standard error of mean. BMI, body mass index; HB, hemoglobin; TLC, total lymphocyte count; ALT, alanine aminotransferase; AST, aspartate aminotransferase; GGT, gamma-glutamyl transpeptidase; ALP, alkaline phosphatase; PT, prothrombin time; INR, international normalized ratio; CR, creatinine; HDL, high-density lipoprotein; LDL, low-density lipoprotein; VLDL, very low density lipoprotein; CAP, controlled attenuation parameter; HOMA-IR, homeostasis model assessment of insulin resistance.

Variable	F 2 and above (N=112)	F 1 and below (N=43)	P-value
Age (years)	46.53±11.75	44.37±12.81	0.321
BMI (kg/m^2^)	27.65±3.42	27.30±3.94	0.587
Waist (cm)	90.7±9.1	90.0±5.9	0.660
Weight (kg)	85.1±14.9	82.3±12.4	0.270
HB (g/dL)	11.9±1.8	13.1±1.6	0.116
TLC (x10^9^/L)	6.0±2.5	6.6±2.5	0.553
Platelets (x10^9^/L)	183.4±84.4	238.4±70.0	0.000
Total bilirubin (mg/dL)	2.2±3.4	0.81±0.51	0.006
ALT (IU/L)	65.2±69.3	51.1±49.0	0.224
AST (U/L)	65.3±68.0	38.8±18.5	0.013
GGT (U/L)	222.9±377.2	103.0±59.2	0.040
ALP (U/L)	143.7±152.6	95.8±33.10	0.044
PT	16.9±4.7	4.5±1.9	0.001
INR	1.4±0.47.0	1.1±0.20	0.001
Albumin (g/dL)	3.4±65.0	3.8±48.0	0.001
CR (mg/dL)	0.96±0.16	0.97±0.29	0.896
Cholesterol (mg/dL)	163.9±62.5	164.1±58.6	0.120
Triglyceride (mg/dL)	169.5±85.3	186.6±118.0	0.985
HDL (mg/dL)	29.5±7.8	30.8±9.5	0.387
LDL (mg/dL)	96.0±20.2	98.3±19.2	0.525
VLDL (mg/dL)	72.3±20.5	71.5±16.7	0.814
Uric acid (mg/dL)	4.7±71.0	4.5±0.54	0.048
CAP (dB/m)	289.3±24.2	287.1±24.9	0.014
Insulin (mIU/L)	10.7±3.1	10.8±2.7	0.909
HOMA-IR	2.7±1.6	5.6±0.68	0.278
Units of alcohol	30.5±14.4	29.1±11.3	0.005

Further multivariant analysis showed that liver fibrosis was correlated with cholesterol level with a significant p-value (p=0.045). Of 28 (18.06%) patients with cirrhosis, six had decompensated cirrhosis, three had hepatocellular carcinoma, and one had acute chronic liver failure. Five patients (3.22%) went on to develop pancreatitis.

## Discussion

The relationship between alcohol consumption and liver disease is evident; however, the question that remains unanswered concerns the relationship between alcohol consumption and the degree of liver damage. It has been observed that the duration of alcohol consumption and the amount of undiluted alcohol intake correlate with the development of cirrhosis. Previous studies have shown that cirrhosis usually develops when lifetime alcohol consumption exceeds 100 kg on undiluted alcohol, which roughly equals to intake of 30 g of undiluted alcohol for 10 years. Alcoholics with daily habits of drinking 80 g of alcohol for the past 10 years are nearly certain to develop liver disease. In a study involving 256 heavy drinkers admitted in the hospital not related to hepatic complaints, liver histology revealed that 45% had steatosis, 34% had steatohepatitis, 10% had developed steatohepatitis with cirrhosis, and 10% had cirrhosis alone [9].

In our study, liver steatosis showed a strong correlation with units of alcohol intake. In northern Italy, a large cohort study conducted by Bellentani et al. corroborated that obese patients with a body mass index (BMI) >25 kg/m^2^ who consumed more than 60 g per day of alcohol were more prone to develop hepatic steatosis, as determined by ultrasonography, in as many as 90% of cases in comparison with their leaner counterparts [9]. In our study, patients with a BMI >23 kg/m^2^ were prone to develop hepatic steatosis associated with insulin resistance as calculated by HOMA-IR (p=0.031).

Previously, it was reported that 40-60 g of undiluted alcohol per day in men and less than 20 g/day for women is safe to consume. However, the study also showed that consuming more than 20 g of pure alcohol increases the chances of liver disease regardless of sex [10].

Many previous studies have concluded that the type of alcoholic beverage bears no correlation with the severity of liver disease [11]. In our study, 98% of patients were consuming whiskey containing 40% to 44% of alcohol; therefore, we could not compare the types of beverages with the severity of liver disease.

ARLD may be acute (e.g., alcoholic hepatitis) or chronic (e.g., steatosis, steatohepatitis, fibrosis, and cirrhosis). The pattern of alcohol consumption also curtails the progression of the disease; drinking with meals as opposed to drinking on an empty stomach lowers the risk of damage to the liver, and binge drinking spares the liver more than continuous alcohol intake [12]. Food affects ethanol absorption, causing a lower peak and slower rise in blood alcohol concentration; however, there are still other yet unknown confounding factors that affect disease severity and risk in different patients with different drinking patterns.

In our study, BMI, insulin resistance, and hyperuricemia, along with the amount of alcohol intake, were associated with ARLD and alcohol-related liver cirrhosis. A longitudinal study was conducted in a population of 13,285 patients to determine the association between self-reported alcohol intake and the risk of developing future liver disease. The study showed a dose-dependent increase in the relative risk of alcohol-induced liver disease above a “threshold” of 14-27 units per week in men and 7-13 units in women [13].

It was observed in the Dionysos Study Group that women had a significantly higher risk than men for any given level of alcohol intake [14]. The study also showed that alcohol-induced liver damage was also increased by drinking on an empty stomach or drinking multiple different varieties of alcoholic beverages, a hypothesis mentioned in other studies as well. In our study, most patients were consuming alcohol before dinner. This observation is also seen in animal experiments, where after a alcoholic binge, ethanol metabolism causes degradation of hepatic mitochondrial DNA and oxidative stress [15].

In the last several decades, alcohol consumption has increased, owing to an increase in economic growth. As a consequence, the incidence of ARLD has increased as well [16]. A recent study performed in China revealed that 70% of alcoholic beverages consumed were spirits, and it is estimated that a staggering 25% of alcohol that is consumed is not even registered [17]. In our study, most patients were consuming whiskey, which was 40% to 44% alcohol by volume. A cross-sectional study revealed that the most consumed alcoholic beverages were homemade alcoholic drinks, such as beer and high-alcohol liquors, in the rural areas of Hunan province [18,19].

Easy access to homemade alcoholic beverages at exceedingly affordable prices has contributed significantly to the high incidence of alcohol consumption in various countries. However, our study cohort used branded alcohol. An Indian study compared the drinking patterns in people with alcohol dependence with and without cirrhosis and collected data on drinking patterns and frequencies. The demographic variables of the study revealed that people in the alcohol-dependent cirrhosis group were older when compared to people with alcohol dependence without cirrhosis (mean age, 45.10 years vs. 39.07 years) [20]. Another Indian study performed in the hospital-based population showed that the age of onset of alcohol usage was 18 years and the age of alcohol dependence was 27 years, compared with the Western population, where the age at first drink and age at dependence are early [21]. In the study by Schneider et al. in 2001, the age at first drink was 15.4±4.7 years, with early onset in males and age at dependence was 22.2±7.9 years [22]. The age of onset of drinking in our study was 18±12.05 years. Narawane et al. and Kamper-Jørgensen et al. found that drinking for more than 14 and 20 years, respectively, was significantly more common in alcoholic liver disease [23,24]. Around 400 g per day was associated with death due to liver cirrhosis related to alcohol [23]. In our country, the minority religions are 5% of the total population. Though most patients in our study are Muslim, minorities are represented out of proportion. They are using alcohol in a larger amount compared to patients who are Muslim. In our country, easily available alcohol is in the form of whiskey and wine.

## Conclusions

In our study, we observed that ARLD is mainly a male-dominant disease. Hypercholesterolemia was common, and it was associated with advance fibrosis of the liver. Most patients in this study consume and/or binge drink highly concentrated alcohol and are alcohol-dependent. Insulin resistance was noticed in a significant number of patients with liver steatosis in ARLD.
